# The cytokine network involved in the host immune response to periodontitis

**DOI:** 10.1038/s41368-019-0064-z

**Published:** 2019-11-05

**Authors:** Weiyi Pan, Qingxuan Wang, Qianming Chen

**Affiliations:** State Key Laboratory of Oral Diseases & National Clinical Research Center for Oral Diseases & Chinese Academy of Medical Sciences Research Unit of Oral Carcinogenesis and Management, West China Hospital of Stomatology, Sichuan University, Chengdu, China

**Keywords:** Periodontitis, Mechanisms of disease

## Abstract

Periodontitis is an inflammatory disease involving the destruction of both soft and hard tissue in the periodontal region. Although dysbiosis of the local microbial community initiates local inflammation, over-activation of the host immune response directly activates osteoclastic activity and alveolar bone loss. Many studies have reported on the cytokine network involved in periodontitis and its crucial and pleiotropic effect on the recruitment of specific immunocytes, control of pathobionts and induction or suppression of osteoclastic activity. Nonetheless, particularities in the stimulation of pathogens in the oral cavity that lead to the specific and complex periodontal cytokine network are far from clarified. Thus, in this review, we begin with an up-to-date aetiological hypothesis of periodontal disease and summarize the roles of cytokines in the host immune response. In addition, we also summarize the latest cytokine-related therapeutic measures for periodontal disease.

## Introduction

Periodontitis is an inflammatory disease indicated by periodontal soft tissue inflammation and the progressive loss of periodontal ligament and alveolar bone.^1^ Soft tissue inflammation, namely, gingivitis, is very common in populations. According to the results of the Fourth National Oral Epidemiological Investigation in China, bleeding on probing was detected in over 85% of adults within 35–64 years of age.^2^ Through a long and slow process, uncontrolled inflammation in the gingiva may lead to the destruction of periodontal tissue and its attachment to teeth, which is defined as periodontitis.^3^ The continuous loss of dentition to support tissue results in tooth looseness and the loss of teeth, which seriously affects patients’ quality of life and causes a tremendous social and economic burden.^4^ Severe periodontitis affects more than 700 million people (11% of the world’s population), making it one of the most prevalent chronic inflammatory diseases worldwide.^4^ In addition, an increasing amount of clinical and experimental evidence indicates the potential direct relationship between periodontitis and several systematic diseases including diabetes, rheumatoid arthritis, atherosclerosis, Alzheimer’s disease and even cancers.^5–9^

The pathogenesis of periodontitis is a problem that plagues investigators. In the 20th century, one or a group of specific microorganisms^10^ were identified as the pathogen of periodontitis by isolation and culture studies. Among these microorganisms, a pathogenic “red complex” that consists of *Porphyromonas gingivalis (P. gingivalis)*, *Treponema denticola* and *Tannerella forsythia* was suggested as the most representative theory of periodontitis pathogenesis in the late 1980s to 1990s.^11,12^ However, with deeper immunological research, the important role of the local host immune response in the pathogenesis of periodontitis was revealed.^13^ In addition, new data obtained from metagenomic and metatranscriptomic studies suggested that a more complicated microbial community is involved in the pathogenesis of periodontitis rather than one or several specific pathogenic bacteria.^14–18^

The initiation and progression of periodontitis are related to multiple aetiologic and risk factors, the most important of which are the local microbiota and host immune response.^19^ Within the progression of periodontitis, the role of cytokines is extremely important. Cytokines are key modulators of both homeostasis and inflammatory processes that act in the first wave of responses against pathogens and stimuli at barrier sites and connect tissue cells with lymphocytes and accessory cell populations.^20^ Many recent studies have found that single nucleoid polymorphisms in cytokines and associated receptor-encoding genes are related to the risk and severity of periodontitis,^21–24^ which indicates that the disordered regulation of cytokines initiates or accelerates periodontitis. On the basis of human studies, studies in experimental animal periodontitis models also found that manipulating the expression of cytokines and their receptors affects the alveolar bone loss phenotype.^25,26^ Research on the cytokine network in periodontal tissue has laid the foundation for the development of cytokine-targeting therapies for periodontal disease, some of which have shown positive effects in pre-clinical trials.^27^ However, compared with the well-discussed site-specific immunocytes and cytokine network in other barrier sites, such as the skin and gastrointestinal and respiratory tracts, how the local immune system in periodontal tissue is trained and activated in healthy and pathological conditions remains to be further explored.^28^ Thus, in this review, we have focused on an up-to-date mechanistic hypothesis of the pathogenesis of periodontal disease and the role of cytokines in periodontal disease. We have also summarized the latest cytokine-related therapeutic measures for periodontal disease.

## The host immune response in periodontitis

**Fig. 1:**
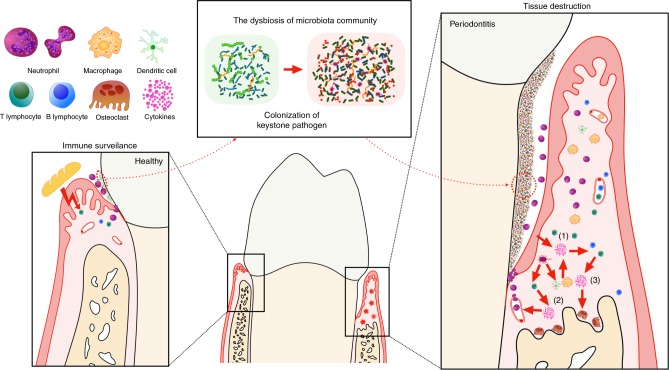
The homeostasis of periodontal tissue, pathogenesis of chronic periodontitis and roles of the involved cytokines. In a healthy state, local challenge and a mild host immune response are balanced. Both the commensal microbiota and mechanical stimulation caused by mastication participate in the training of local mucosal immunity. In this state, there is an appropriate number of infiltrating neutrophils in the gingival sulcus, as well as some resident immune cells in the gingival tissue, including Th17 cells and innate lymphoid cells. However, if the immune pathogenicity of the local microbiota is elevated by the colonization of keystone pathogens, which over-activate the host immune response, tissue destruction is initiated. The interaction between the microbiota and all host cells leads to the first wave of cytokine secretion (1), which mainly participates in the amplification of the pro-inflammatory cytokine cascade and the recruitment, activation and differentiation of specific immune cells. In addition, a group of cytokines (2) closely related to the differentiation of a specific subset of lymphocytes are secreted by MNPs and APCs after stimulation by the microbiome. Each of these cell subsets secretes a certain pattern of cytokines, which might act as the positive-feedback factor or direct effector (3), eventually leading to tissue destruction

As with other barrier sites, such as the gastrointestinal and respiratory tracts, the periodontal tissue is continuously exposed to the oral microbiota and other physical and chemical stimuli generated by mastication and respiration.^28^ There exists a delicate balance between the local immune response and the microbiota in physiological conditions. Immune surveillance and toleration of the local microbiota are achieved without a severe inflammatory response^29^ (Fig. 1, left side). Nevertheless, after the colonization of a “keystone” pathogen, the constituents of the microbiota and their total counts are altered, which elevates the pathogenicity of the whole community and disrupts tissue homeostasis^30^ (Fig. 1, middle). Under these conditions, the immune response is over-activated, which leads to the infiltration of immune cells, activation of osteoclastic activity, and eventually the destruction of both soft and hard tissue (Fig. 1, right side).

The pathological host immune response against local dysbiotic microbes can categorized roughly into three stages (Fig. 1, right side). The first wave of challenge occurs directly between the microbiome and host cells that include periodontal tissue cells, namely, mucosal epithelial cells and gingival fibroblasts, and other immunocytes. In addition, because of the continuous stimulation and damage caused by the local microbiome and mastication, immune cells such as mononuclear phagocytes (MNPs), antigen-presenting cells (APCs) and specific T cell subsets (such as type 17 helper T cells, Th17 cells) reside locally and can be recruited. The interactions between the microbiome and all host cells lead to the first wave of cytokine secretion (1) by activation of the pattern recognition receptor and its downstream signalling. Representatives of these cytokines are the interleukin-1 (IL-1) family, the IL-6 family and tumour necrosis factor (TNF). These cytokines have pleiotropic effects on lymphocyte promotion and tissue destruction and are all recognized as pro-inflammatory cytokines. In addition, a group of cytokines (2) closely related to the differentiation of a specific subset of lymphocytes are secreted by MNPs, APCs and local lymphocytes after stimulation by the microbiome. With the participation of members from the IL-1 and IL-6 families, the stimulation of these cytokines leads to activation of the corresponding signalling pathways and the maturation and differentiation of certain cells. Each of these cell subsets secrete a certain pattern of cytokines, which might act as a positive feedback mechanism or direct effector (3). Most of the effects of these cytokines and cell subsets are extremely complicated, including enhancement of the mucosal barrier, control of pathobionts, induction or suppression of osteoclastic activity and feedback inhibition of the over-activated immune response.

The relationship between immune surveillance and the oral microbe-induced host immune response can be diverse and different. If local stimulation and a mild host immune response are balanced, immunological surveillance and an appropriate immune response dominate.^28^ However, if the pathogenicity of the local microbiota is elevated by the colonization of keystone pathogens that over-activate the host immune response, tissue destruction is initiated. However, the pathogenesis and immune response to periodontitis are far from clear. The model of Th1 vs. Th2 cells suggested elsewhere was proven to be unsuitable in periodontitis because of contradictory results from studies of this theory,^31^ which has driven investigators to explore a more tissue-specific mode of the periodontal host immune response within periodontitis. Some new participants in the cytokine and immunocyte network involved in periodontitis were recently reported.

**Fig. 2:**
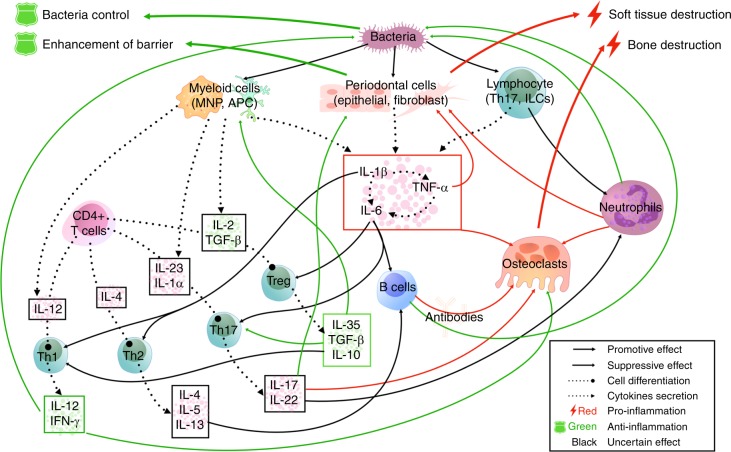
The cytokine network in the pathogenesis of periodontitis. In this figure, the effects of cytokines in the host immune response are shown at the level of intercellular interactions. Briefly, well-established pro-inflammatory cytokines from IL-1, IL-6 and TNF families are secreted by host periodontal cells and immunocytes after stimulation by pathobionts, which activates and recruits specific immune cell subsets and causes direct tissue damage. Then, naive T cells and B cells differentiate into mature T cells or plasma cells under the action of specific cytokines and further activate or promote other effector cells, such as osteoclasts and neutrophils, which exert pro-inflammatory or anti-inflammatory effects by secreting cell-specific cytokine clusters. Among these cell subsets, Th1 and Treg cells mainly act as protectors, while Th2/B and Th17 cells exert complex effects that may lead to tissue destruction or protection under certain circumstances (full lines: the effect of cytokines on cells and the interactions between cells; dashed lines: the secretion of cytokines)

Generally, the classification of cytokines is established by structural similarity, gene homology and the sharing of receptors. To clarify cytokine classification, we will discuss the cytokine network involved in periodontitis following this three-stage method of cytokine classification. First, we discuss well-established pro-inflammatory cytokines including members of the IL-1 family, IL-6 family and TNF family and some recently identified members and their functions. Second, cytokines are closely associated with certain cell subsets, including Th1 cell-, Th2 cell-, Th17 cell- and Treg (regulatory T) cell-related cytokines. These cytokines and their related cells form a complex and integrated host immune response network against bacteria that is summarized in Fig. 2. The function of each cytokine is described in detail below. Finally, some additional anti-inflammatory cytokines and their roles in periodontitis are reviewed.

## Pro-inflammatory cytokines involved in periodontitis

**Fig. 3:**
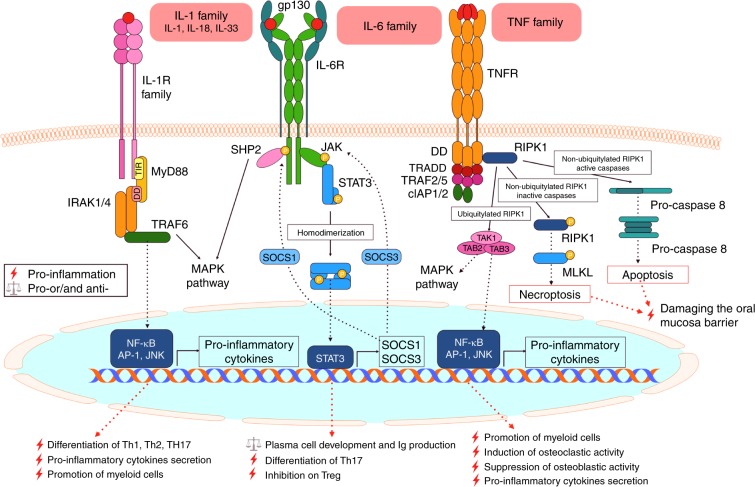
Pro-inflammatory cytokines, related receptor complexes and downstream signalling pathways. Most IL-1 (represented by IL-1, IL-18 and IL-33), IL-6 and TNF family members have pleiotropic effects on lymphocyte promotion and tissue destruction and act as pro-inflammatory cytokines. By binding to their corresponding receptor, IL-1 family members mainly activate transcription factors related to T cell activation and pro-inflammatory cytokine secretion, and IL-6 mainly mediates B cell activation. Depending on the state of key transduction proteins, the binding between TNF family members and their related receptors can lead to very different cell fates that include death (apoptosis and necroptosis) or life (secretion of pro-inflammatory and osteoclastogenic factors) and both lead to the destruction of periodontal tissue

The well-established pro-inflammatory cytokines (members of the IL-1, IL-6 and TNF families) are reviewed first. The related receptors, downstream signalling pathways and functions of these cytokines are summarized in Fig. 3.

### IL-1 family

IL-1, which was discovered and named 40 years ago, is part of a complex family of cytokines with members found by gene cloning and molecular identification; these IL-1 family members include IL-1α, IL-1β, IL-18, IL-33, IL-36, IL-37 and IL-38. IL-1 family members are involved in both innate and adaptive immune responses and were proven to be related to inflammation, autoimmunity, cardiovascular disorders and cancer.^32^ The IL-1 family is composed of 11 molecules and 10 associated receptors.^32^ The binding between ligands and dimeric receptors recruits the adaptor protein myeloid differentiation factor-88 (Myd88) through a shared intracellular signalling domain named the Toll-IL-1 resistance (TIR) domain. This binding further activates downstream proteins including IL-1R-associated kinases (IRAKs) and tumour necrosis factor receptor-associated factor 6 (TRAF6), leading to the activation of inflammatory-related transcriptional factors including nuclear factor-κB (NF-κB), activator protein-1 (AP-1) and c-Jun N-terminal kinase (JNK).^33^

Among IL-1 family members, IL-1, IL-18 and IL-33 are regarded as drivers of the differentiation and polarization of myeloid cells and lymphoid cells under microbial or environmental challenge. The role of these cytokines in the pathogenesis of periodontitis has been studied thoroughly. However, IL-37 is recognized as an anti-inflammatory cytokine^34^ with a negative regulatory effect against inflammation and is discussed in the next section.

IL-1 and IL-33 are both categorized in the IL-1 subfamily because of their structural similarity. A large number of clinical studies and systematic reviews have demonstrated that gene polymorphisms in *IL-1B, IL-1R* (which encodes the IL-1-associated receptor) and *IL-1N* (which encodes the receptor antagonist IL-1Ra) are related to a susceptibility to periodontitis,^35^ which indicates that IL-1β is involved in the pathogenesis of periodontitis. IL-1β was shown to be induced upon host-microbiota interaction and broadly induces the expansion and activation of both Th1 and Th2 cells.^36^ In addition, IL-1, especially IL-1α, drives type 3 immunity through assisting IL-23 and IL-6 in the activation of Th17 cells^37,38^ and the expression of IL-17. The total amount of IL-1β in gingival crevicular fluid (GCF), which has been shown to be related to the severity of periodontitis,^39^ also decreases after non-surgical periodontal therapy (NSPT).^40^ Moreover, activation of the NLRP3 inflammasome, which mediates the cleavage of pro-IL-1β into its active form, was also observed in the periodontal tissue of periodontitis patients.^41,42^

Due to the differential expression of receptors and regulatory molecules in target cells, the functions of different IL-1 family members are specific. IL-33 was shown to be secreted by both haematopoietic and parenchymal cells and involved in the modulation of type 2 innate lymphoid cells (ILC2) and Th2 cells, which are mainly related to type 2 immunity.^32^ The high-level expression of IL-33 was observed in the periodontal tissue of chronic periodontitis patients.^43^ However, whether the amount of IL-33 is increased in the GCF of chronic periodontitis patients is controversial.^44–47^ According to in vitro and in vivo animal experiments, the expression of IL-33 can be induced by gingipain, fimbriae and lipopeptide from *P. gingivalis*^48,49^ and might lead to alveolar bone destruction through a receptor activator of nuclear factor-κB ligand (RANKL)-dependent pathway.^50,51^

IL-18 was first recognized as an interferon (IFN)-γ-inducing factor and an activator of NK (natural killer) cells and Th1 cells, which are closely related to type 1 immunity.^52^ Polymorphisms were identified in the promoter of IL-18 and also shown to be related to the increased risk of periodontitis.^23,24^ In addition, the upregulation of IL-18 was detected in the GCF,^53^ saliva,^54^ and serum^55^ of chronic periodontitis patients, and IL-18 levels decreased after NSPT.^56–58^ Active *P. gingivalis* and its lipopolysaccharide (LPS) were shown to upregulate IL-18 expression.^59,60^ Furthermore, IL-18 was shown to stimulate the expression of matrix metalloproteinase,^61^ and the overexpression of IL-18 leads to inflammatory bone loss after oral bacterial infection.^62^

### IL-6 family

Since IL-6 was named and identified nearly 30 years ago,^63^ research on IL-6 has identified multiple members of the IL-6 family and determined their pleiotropic functions in the immune response, haematopoiesis, early development, bone metabolism and cancer.^64,65^ The IL-6 family consists of 10 members identified according to their common use of receptor chain gp130 (also known as CD130).^66^ Among member of the IL-6 family, IL-6 was shown to be related to the risk and pathogenesis of periodontitis, while IL-11 and the recently added member IL-35^67^ seem to act as anti-inflammatory cytokines and are discussed in the next section. The receptor to which IL-6 directly binds is IL-6R, which is expressed in a transmembrane form (mIL-6R) in cells such as hepatocytes, monocytes and lymphocytes.^68^ IL-6R can also exist in a soluble form (aIL-6R) after the cleavage of mIL-6R by the proteases ADAM-10 (a disintegrin and metalloproteinase domain-containing protein 10) and ADAM-17.^69^ However, since IL-6R lacks an intracellular signalling transduction domain, a second signal transducer, the ubiquitously expressed transmembrane protein gp130, is needed to activate downstream signalling transduction.^70^

The source of IL-6R determines the mode of IL-6 signalling pathway activation; the IL-6 signalling pathway can be activated by classic signalling (mIL-6R), trans-signalling (sIL-6R) and trans-presentation (mIL-6R in neighbouring cells).^71^ After formation of the IL-6-IL-6R-gp130 complex, Janus kinase (JAK) is recruited and mediates the phosphorylation of signal transducer and activator of transcription 3 (STAT3) and the formation of phosphorylated STAT3 homodimers. Activation of the JAK-STAT3 signalling pathway upregulates the expression of IL-6-responsive genes including suppressor of cytokine signalling 1 (SOCS1) and SOCS3.^72^ In addition, JAK phosphorylates the cytoplasmic domain of gp130 at tyrosine 759 (Y759), which acts as the binding site of SH2 domain tyrosine phosphatase 2 (SHP2), further activating the mitogen-activated protein kinase (MAPK) pathway.^73^ SOCS1 and SOCS3 are able to directly bind activated JAK and JAK phosphorylated at Y759, which acts as a negative feedback loop against the JAK-STAT3 and MAPK pathways, respectively.

IL-6 is secreted from many types of activated immune cells, including dendritic cells (DCs), macrophages, B cells and T cells, and non-immune cells, including fibroblasts, keratinocytes and endothelial cells. The transcription of IL-6 can be regulated by multiple transcription factors including AP-1, NF-kB, C/EBPβ and cAMP-responsive elements. NF-kB efficiently induces IL-6 transcription after activation by LPS, IL-1, IL-17 and tumour necrosis factor-α (TNF-α).^74^ IL-6 has been well characterized as a major player in chronic inflammation and was proven to have multiple functions in many cells. Moreover, IL-6 was shown to be related to plasma cell development^75^ and efficiently induce the immunoglobulin production.^76^ In addition, IL-6 trans-presentation in mIL-6R-expressing DCs induces the Th17 cell-induced differentiation of gp130-expressing CD4^+^ T cells,^77^ whereas IL-6 inhibits the differentiation of Treg cells.^78,79^ Taken together, these results indicate that IL-6 is an inflammatory amplifier induced by pathogen-associated molecular patterns, stimuli and pro-inflammatory cytokines and that IL-6 also has a pro-inflammatory effect on the adaptive immune response.

The involvement of IL-6 in periodontitis is well recognized. The IL-6 174G/C polymorphism was shown to be associated with susceptibility to chronic periodontitis by a meta-analysis including 21 studies.^80^ An increased amount of IL-6 in the GCF of chronic periodontitis patients was also demonstrated by a comprehensive meta-analysis, while no significant decrease in IL-6 levels was found after NSPT.^81^ The continuous observation of IL-6 levels in an experimental periodontitis monkey model also revealed that the expression of IL-6 is induced in the initiation phase but remains low in the progression and resolution phases of periodontitis.^82^ These results indicated that IL-6 plays a crucial role mainly in the initiation and acute phase of periodontitis. In addition to its function in the immune response, IL-6 was reported to participate in bone homeostasis. The expression of the receptor activator of RANKL in osteoblasts was upregulated by IL-6, which led to the differentiation of osteoclasts and bone resorption.^83,84^ Because of its pathogen-related upstream regulation, broad derivation from multiple cell groups and direct effect on immune response and osteoclastic activity, IL-6 was applied widely as a representative pro-inflammatory and damage-related cytokines in in vitro studies and in vivo animal studies and shown to be upregulated after stimulation and downregulated after treatment.^85–89^

### TNF family

TNF was first recognized as a tumour necrotizing substance and named in 1975.^90^ The two TNF family members, TNF-α and TNF-β, were purified and identified in the 1980s and share the same membrane receptor.^91–93^ In addition to its induction of cell death, TNF acts as a crucial player in the pro-inflammatory response and cellular communication.^94^ TNF was initially expressed as a type II transmembrane protein and shown to exist as a homotrimer that is cleaved by ADAM-17 into a soluble form.^95–98^ Soluble TNF is able to bind both TNFR1 and TNFR2, the two best characterized receptors of TNF family members, and activate downstream transcriptional factors represented by NF-kB and JNK through a distinct signalling cascade. TNFR1 is widely expressed in most mammalian cells, while the expression of TNFR2 is restricted to immune and endothelial cells.^99^ The binding of TNF to the TNF receptor TNFR1 recruits the TNFR1-associated death domain protein (TRADD) through its cytoplasmic death domain (DD), which further leads to the formation of complex I with receptor-interacting serine/threonine-protein kinase 1 (RIPK1).^100,101^ Then, a group of proteins including TRAF2/5, cellular inhibitor of apoptosis protein 1 (cIAP1) and linear ubiquitin chain assembly complex (LUBAC) is recruited to complex I. These proteins are all related to the ubiquitylation status of RIPK1, which determines the downstream signalling pathway and cell fate.^102,103^ Since TNFR2 lacks a DD, it directly recruits TRAF1/2, leads to the formation of complex I as well.^104^

As for downstream signalling, if RIPK1 is ubiquitylated, the ubiquitin chain attached to RIPK1 recruits the TAK1/TAB complex (TGFβ-activated kinase 1 and the TAK1 and MAP3K7-binding protein), which further activates the NF-kB, JNK and p38 signalling pathway.^105–110^ However, if RIPK1 is not ubiquitylated, pathways leading to different types of cell death are activated. Un-ubiquitylated RIPK1 is released from complex I and initiates the assembly of complex II, which leads to apoptosis with active caspase-8 (complex IIa and IIb)^111,112^ and necroptosis with inactive caspase and the participation of RIPK3 and mixed lineage kinase domain-like (MLKL) (complex IIc).^113–115^ In addition, TNF was shown to participate in bone metabolism. Although TNF does not induce osteoclast differentiation directly, it induces the expression of RANK in osteoclast precursors and RANKL in osteoblasts.^116–119^ Nevertheless, TNF also downregulates the expression of osterix (OSX) and runt-related transcription factor 2 (RUNX2) in osteoblasts.^120,121^ Taken together, these results suggest that TNF exacerbates bone resorption by increasing osteoclastic activity and deceasing osteoblastic activity.

TNF was shown to participate in the pathogenesis of periodontitis. Several polymorphisms in the promoter region of TNF-α have been identified, and the 308G/A and 863C/A polymorphisms may contribute to the susceptibility of periodontitis according to a meta-analysis.^22^ The TNF level was also shown to be elevated in the GCF^122^ and serum^123^ of chronic periodontitis patients, while no decrease in TNF was observed after NSPT. These results can be explained by the participation of TNF in bone metabolism, and studies have shown that TNF upregulates RANKL expression in gingival epithelial cells,^124,125^ T cells and osteoblasts. Interestingly, it was reported that TNF mediates the apoptosis^126^ of gingival fibroblasts and epithelial cells and inhibits extracellular matrix production in gingival fibroblasts,^127^ indicating that TNF might be involved in the initiation of periodontitis by damaging the oral mucosa barrier. Moreover, it is notable that due to the participation of circulating TNF in the pathogenesis of other systematic diseases, a high level of circulating TNF potentially links periodontitis with diabetes^128^ and rheumatoid arthritis^129^ by contributing to the systemic inflammatory burden.

## Cytokines related to specific types of immune cells

**Fig. 4:**
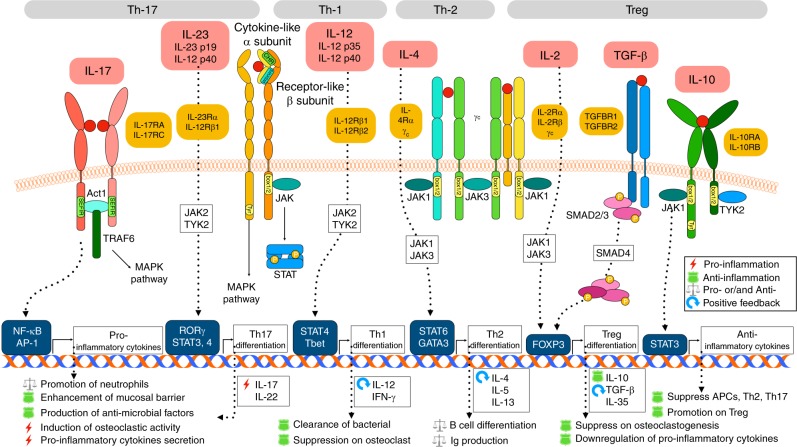
Cytokines that are closely related to certain groups of T lymphocytes. Most of the remaining cytokines are closely related to the differentiation and/or effects of specific immune cell subsets. Under stimulation by certain inflammatory cytokines, naive CD4^+^ T cells differentiate towards multiple directions, including Th1 (IL-12) and Treg (IL-2 and TGF-β) cells, which mainly have protective effects, and Th17 (IL-23) and Th2 (IL-4) cells, which mainly have pleiotropic effects. The signalling pathways downstream of IL-17 (secreted by Th17 cells) and IL-10 (secreted by Treg cells) are specific and of special significance to the periodontal host immune response, as shown in this figure

The most representative cytokines closely associated with the cell subsets Th1, Th2, Th17 and Treg cells are now reviewed. The related receptors, downstream signalling pathways and functions of these cytokines are summarized in Fig. 4.

### Th1 cells: IL-12, IFN-γ

The IL-12 family is a group of cytokines that are structurally related to IL-6 family members including IL-12, IL-23 and IL-27.^130^ The binding of IL-12 with its receptor is based on the formation of a heterodimer of two subunits, which is similar to the binding of IL-6 and sIL-6Rα. Briefly, a heterodimer consisting of a cytokine-like α subunit (IL-12 p35, IL-23 p19 and IL-27 p28) and a receptor-like β subunit (IL-12 p40 for both IL-12 and IL-23, Epstein-Barr virus-induced gene 3 for IL-27) binds to a group of receptors (similar to the binding of IL-6 and gp130), which activates the downstream JAK-STAT signalling pathway.^131–133^ Among these cytokines, IL-23 is closely associated with Th17 cells, and IL-27 exerted a potential protective effect in the immune response to periodontitis, which is discussed later.

IL-12 is secreted by MNPs and dendritic cells under pathogen stimulation and exhibits the strong ability to promote IFN-γ production in T and NK cells.^134^ Naive CD4^+^ T cells differentiate into Th1 cells in the presence of IL-12 with activation of the transcription factors STAT-4 and T-bet and secrete a group of cytokines including IL-12 (as positive feedback)^135^ and IFN-γ for bacterial clearance.^136^ While the Th1 response may occur during the establishment of inflammation, it may also act to protect against tissue destruction. The amount of IL-12 in the GCF of chronic periodontitis patients was similar to that in healthy controls^81^ and increased after NSPT.^137,138^ In addition, IL-12 and IFN-γ were found to be negatively correlated with the gingival sulcular depth.^139^ According to animal and in vi*tro* studies, IL-12 p40(−/−) mice exhibit increased tissue destruction after *P. gingivalis* infection.^25^ The application of antibodies against IL-12 p35 and IL-12 p40 had a similar effect.^113^ In addition, early findings showed that IL-12 and IFN-γ inhibit osteoclastogenesis and bone resorption^140,141^ in vitro, and IL-12 is not involved in infection-induced bone resorption in vivo.^142^ Taken together, these results suggest that IL-12, IFN-γ and, presumably, Th1 cells mediate the clearance of local bacteria and control of *P. gingivalis*, suppress osteoclastic activity, and exhibit mainly protective effects in the pathogenesis of periodontitis.

### Th2 cells: IL-4

IL-4 is a member of the γ_c_ family, the members of which, including IL-2, IL-4, IL-7, IL-9, IL-15 and IL-21, share a common cytokine receptor γ chain.^143^ Most γ_c_ family members bind a heterodimeric receptor complex formed by γ_c_ and another type I cytokine receptor,^144^ while IL-2 and IL-15 require one more sushi domain-containing receptor to form a heterotrimeric complex.^145,146^ Interestingly, all the receptors of γ_c_ family cytokines activate JAK1 and JAK3 (all by γ_c_) after cytokine binding and further activate different kinds of STAT. IL-4 is mainly secreted by naive CD4^+^ T cells and B cells, which mainly activates STAT6 and GATA3, mediates Th2 cell^147^ and B cell^148^ differentiation and initiates type II immunity. Activated Th2 cells mainly secrete IL-4, IL-5 and IL-13 to mediate the humoural response. As mentioned above, the Th1 cell vs. Th2 cell hypothesis might not be a suitable model to explain the mode of periodontitis pathogenesis, and the role of Th2 cells and type II immunity in periodontitis shows this complexity. On the one hand, IL-4 was determined by meta-analysis to be the only cytokine decreased in chronic periodontitis patients and elevated after periodontal treatment,^81^ which indicates that IL-4 and Th2 cells have protective effects in periodontitis. In addition, it was reported that IL-4 is able to downregulate pro-inflammatory cytokines^149^ and restrain osteoclastogenesis.^3^ On the other hand, Th2 cells also support the destructive B cell response,^150,151^ and the infiltration of abundant B cells and plasma cells, a sign of type II immunity, was observed in progressed periodontitis.^151^ This inconsistency in data and results has driven investigators to search for other hidden mechanisms to explain the mode of periodontitis pathogenesis.

### Th17 cells: IL-23 and IL-17

IL-17A, commonly known as IL-17, and its binding receptor IL-17RA were discovered in the middle 1990s,^152,153^ which was found upregulated in human inflammatory and autoimmune disease.^154,155^ The source of IL-17 could not be determined in any cluster of lymphocytes known at the time until the discovery of IL-17-secreting Th17 cells ten years later. Th17 cells, which are activated by the IL-12 family member IL-23 as mentioned above, characteristically express RAR-related orphan receptor gamma (RORγ).^156^

As mentioned above, IL-23 is a member of IL-12 family, and the activation of its downstream signalling requires the participation of the IL-12 p40 subunit and a heterodimeric receptor consisting of IL-12Rβ1 and IL-23Rα.^131,157^ In addition to the abovementioned effects of IL-6 and IL-1 on the differentiation of Th17 cells, IL-23 was more particular in promoting the pathogenicity of Th17 cells. The mechanism by which this effect occurs includes the induction of pathogenicity-related gene (IL-23R,^31,158^ TGF-β3^158^) expression and the suppression of anti-inflammatory IL-10.^159^ IL-23 was secreted by myeloid APCs exposed to *P. gingivalis*^160,161^ and periodontal ligament fibroblasts stimulated by IL-1β.^161^ The correlation between the amount of IL-23 in the GCF and attachment loss was also determined in chronic periodontitis patients.^162^

The IL-17 family consist of six family members, IL-17A to IL-17F, identified by screening for homologous genes,^163^ and IL-17E was now known as IL-25 due to its distinct function closely associated with type II immunity^164^ and airway allergic disease.^165,166^ The family of IL-17 receptors shares a cytoplasmic motif named “SEFIR” (similar expression to fibroblast growth factor-IL-1 receptor) by which IL-17R recruits NF-κB activator 1 (Act1) after the binding of IL-17.^167^ Act1 then recruits and ubiquitinates TRAF6 through its E3 ubiquitin ligase activity,^168,169^ triggering downstream transcriptional factors including NF-κB and AP-1;^170^ this process is similar to signalling downstream of IL-1 and TNF-α. Among the IL-17 family members, IL-17A and IL-17F share the same receptor complex^171^ (a heterodimer of IL-17RA and IL-17RC subunits) and have similar functions in immune surveillance at mucosal and barrier surfaces^172^ and the progression of chronic inflammation.^173^ In addition to Th17 cells, other subset of lymphocytes can also secret IL-17; these include γδ T cells, ILC3s and NK cells.^174^ The functions of IL-17 in oral immunity and the microbiome has been well reviewed elsewhere. Briefly, these functions can be categorized into three features: the generation and recruitment of neutrophils, the upregulation of antimicrobial factor expression, and exertion of a protective effect on the local mucosal barrier.^175^ These functions are all closely related to periodontal barrier integrity and control of the oral microbiota. In addition, IL-17 plays an important role in protecting the host from *Candida albicans* infection.^176,177^ Interestingly, although IL-17 has shown the protective function above, Th17 cell-secreted IL-17 is also closely related to pathogenesis of periodontitis. Elevated IL-17 levels were determined in the GCF of chronic periodontitis patients^178^ and positively correlated with disease severity.^160,179^ It was also reported that IL-17 and a dysbiotic microbiome might promote each other, leading to the enhancement of both microbiome pathogenicity and mucosal immunopathology.^180,181^ In addition, developmental endothelial locus-1 (DEL-1), a protein that mainly affects the adhesion and transmigration of neutrophils on the vascular endothelium,^182^ showed antagonistic effect against IL-17 in the recruitment of neutrophils.^183^ A number of informative studies published recently reported the protective effect of DEL-1 against local inflammation and bone loss.^26,184,185^ In conclusion, IL-17 plays important roles in both local tissue homeostasis and the pathogenesis of periodontitis. The so-called “immune plasticity” regulated by the balance between IL-17 and its antergic factors such as DEL-1 determines the effect of the IL-23-IL-17 axis on pathogenesis.

### Treg cells: IL-2, TGF-β, and the IL-10 family

IL-2 was the first identified member of the γ_c_ family shown to be a T cell growth factor in 1976.^186^ The activation of IL-2 signalling requires the formation of a heterotrimeric receptor complex (IL-2Rα, IL-2Rβ and γ_c_)^187^ that recruits JAK1 and JAK3 and activates STAT signalling mediated by STAT5.^188^ IL-2 possess multiple pleiotropic functions, including promoting T cell and NK cell proliferation and B cell and T cell (except for Th17 and T follicular helper cells) differentiation.^189^ The most distinct characteristic of IL-2 is its promoting effect on Treg cells.^143^ The expression of IL-2 in periodontitis patients was reported to be similar with that in controls^82^ or even decreased^190^ in serum, and an animal study showed that the expression of IL-2 is elevated in the resolution stage of experimental periodontitis.^82^ These results suggest that IL-2 acts as an inhibitory factor in the development of periodontitis.

The TGF-β (transforming growth factor-β) subfamily belongs to a huge superfamily containing 32 members, among which TGF-β is the most relevant member for immune regulation.^191^ TGF-β is synthesized as a pro-hormone that requires cleavage by both intercellular and extracellular proteases^192^ to be activated. The receptor complex of TGF-β is a paired kinase receptor that mediates the phosphorylation of SMAD2/3, following which a heterotrimeric transcriptional complex consisting of phosphorylated SMAD2, SMAD3 and SMAD4 is formed and affects numerous downstream pathways by interacting with other transcription factors.^193,194^ Synergy between IL-2 and TGF-β triggers forkhead box P3 (FOXP3) expression in naive CD4^+^ T cells, which leads to the differentiation of Treg cells.^195^ The most representative cytokines secreted by Treg cells are TGF-β, which acts as a positive feedback loop, and IL-10.

IL-10, the first recognized cytokine in the IL-10 family, is the only member of the IL-10 subfamily with its special functions.^196^ After IL-10 binds to a heterodimeric receptor (IL-10RA and IL-10RB),^197^ the intercellular region of the receptor complex recruits JAK1 and tyrosine kinase 2 (TYK2) and thereafter activates STAT signalling mediated by STAT3.^196^ IL-10 has a broad suppressive effect on APCs,^198^ Th2 cells,^199^ and Th17^200^ cells and the production of pro-inflammatory cytokines and a stimulating effect on Treg cells.^201,202^ In addition, IL-10 was reported to be able to suppress RANKL expression in activated T cells.^203,204^

Because TGF-β and IL-10 are the main effector cytokines in Treg cells, their roles in periodontitis will be discussed jointly. According to the detection of increased FOXP3^+^ Treg cells in inflamed periodontal tissue,^205–208^ as well as an increase in Treg cells after the infiltration of activated T cells,^207^ Treg cells may exert regulatory and protective effects against overactivated inflammation. Interestingly, the role of Treg cells in the maintenance of periodontal homeostasis is not as significant as their role in other barrier sites.^209^ The reason for this finding may relate to the continuous activation of the periodontal host immune response, which drives the differentiation of CD4^+^ T cells towards Th17 cells rather than Treg cells. The crucial role of Th17 in periodontal homeostasis discussed above also supports this inference.

## Other anti-inflammatory cytokines involved in periodontitis

In addition to Treg cell-related cytokines, some other cytokines have anti-inflammatory effects in periodontitis. IL-11 is a member of the IL-6 family that also activates JAK/STAT3 signalling pathway through its binding to the IL-11Rα-gp130 complex.^66^ IL-11 regulates the polarization of T cells^210^ and downregulates pro-inflammatory cytokines, which are protective factors in other inflammatory diseases.^211,212^ Many studies have shown IL-11 to be a protective cytokine and calculated the IL-11/IL-17 or IL-11/IL-1β ratios, which indicate the balance between pro- and anti-inflammatory cytokines. The results of these studies showed that these ratios are decreased in chronic and aggressive periodontitis patients.^213–216^ In addition, a pre-clinical animal study determined the therapeutic effect of recombinant IL-11 on ligature-induced periodontal disease in the beagle.^27^

IL-27, a member of the IL-12 family, is a heterodimeric cytokine consisting of two subunits: IL-27 p28 (also known as IL-30) and Epstein-Barr virus-induced molecule 3 (EBI3).^217^ IL-27 was first found to promote the Th1 response by activating IFN-γ secretion in CD4^+^ T cells and NK cells.^218^ However, studies in IL-27-Rα-deficient mice determined that IL-27 is not required for IFN-γ production.^219^ A number of studies then reported that IL-27 successively inhibited the immunopathology of the Th17 response^220–222^ and GM-CSF production in T cells.^223,224^ In addition, IL-27 induced the expression of the anti-inflammatory cytokine IL-10 in Th1, Th2, and Th17 cells.^225–227^ Only a few articles have investigated IL-27 in the periodontal region; these studies showed that IL-27 might not be highly expressed in the GCF of chronic periodontitis patients^228^ but is upregulated in both GCF and serum after periodontal treatment.^229,230^ These clinical data indicated that IL-27 is likely an anti-inflammatory cytokine that participates mainly in the resolution or steady stage of periodontitis.

As mentioned above, IL-37 is a newly recognized IL-1 family member that binds IL-1R5 (also known as IL-18Ra) and the IL-1R8 receptor complex.^231^ It was reported that IL-37 secreted from Treg cells suppresses the function of NK cells,^232^ indicating that IL-37 is an anti-inflammatory cytokine. Interestingly, recently published studies reported that IL-37 can be secreted by a certain subset of plasma cells and suppress osteoclast formation in vitro.^233^ Clinical data also demonstrated the downregulation of IL-37 in the GCF of chronic periodontitis patients.^44^ In addition, a large-scale genome-wide association study determined a polymorphism (rs3811046) in the IL-37 locus associated with the high-level expression of IL-1β in GCF.^234^ All the evidence reported so far shows that IL-37 might be an anti-inflammatory cytokine.

## Cytokine-targeted therapies for periodontitis

The tissue destruction observed in many other chronic inflammatory diseases, such as rheumatoid arthritis,^235^ inflammatory bowel disease,^236^ ankylosing spondylitis,^237^ psoriasis^238^ and asthma,^239^ is also mediated by the host immune response. With an increasing understanding of the immune response in the initiation and progression of disease, the identification of cytokines with certain functions generally leads to the development of cytokine-targeting therapies.^240^ These therapies mainly block targeted pathogenic cytokines using monoclonal or polyclonal antibodies or introduce protective anti-inflammatory agents, such as anti-inflammatory cytokines, receptor antagonists, and decoy receptors. In contrast, according to the currently published literature, few cytokine-targeted therapies have been developed for chronic periodontitis. Published clinical trials were all small-sized and assessed the therapeutic effect of cytokine-targeting therapy on other diseases closely associated with periodontitis, such as rheumatoid arthritis. In these studies, the periodontal examination was performed incidentally, and the results are inconsistent.^241–244^ In addition, a few studies reported a pre-clinical assessment of cytokine-targeting therapy using experimental periodontitis models^27,245–247^ in animals such as primates, dogs, mice and rats. The blocking antibody, antagonist and anti-inflammatory cytokines all had inhibitory effects on inflammatory bone resorption. The lack of cytokine-targeted therapies may be related to the satisfactory therapeutic effect of basic non-surgical periodontal therapy, which removes the pathogenic microbiota community simply and efficiently. However, for patients who are highly susceptible to periodontitis and suffer from systemic inflammatory diseases closely related to periodontitis, we believe that well-developed cytokine-targeted therapies exert irreplaceable effects. Along with a deepening understanding of periodontal host immunity and the cytokine network, more tissue-specific responses worthy of the development of targeted therapies will be identified.

## Concluding remarks

As mentioned above, studies aimed at understanding periodontal host immunity and the involved cytokine network have reported surprising results. The most intriguing among these results is the crucial role of Th17 cells and their related cytokines in periodontal tissue-specific immunity. These findings remind us that the specific environment around periodontal tissue leads to the specific mode of homeostasis in healthy individuals and the pathogenesis of periodontitis. In addition, with the rapid development of single cell technology, an enormous amount of information that we have never been exposed to has flooded into the field of immunology, including many newly identified immunocyte subsets and cytokines whose functions in periodontal tissue have not been determined. All of these findings remind us that the immune response patterns and cytokine networks in periodontal tissue in both healthy and inflammatory conditions are far from clear. In addition, due to the complexity of periodontal microbiota immunity, studies aimed at a single microbial species may contribute less to understanding the host-microbiome interaction. Therefore, tissue-specific host immunity may be the future of research on the pathogenesis of periodontitis. Based on a longstanding understanding of periodontal histopathology, neutrophils play crucial roles in both tissue homeostasis and periodontitis pathogenesis. In addition, Th17 cells were recently found to play a major role in the recruitment of neutrophils. Therefore, a good direction for future research may be aimed at newly identified immune cell subsets and cytokines that support neutrophils and Th17 cells. In addition, most published studies have focused on pro-inflammatory cytokines and pathogenic cell subsets. However, the functions of the most classic pathogenic Th1 and Th2 cells and their associated cytokines have not been defined and may not be easy to define as pro- or anti-inflammatory in the future, which also brings difficulties for the development of cytokine-targeting therapies. However, studies on inhibitory cytokines and cell populations might help break this impasse. Some pre-clinical studies have shown the protective effect of inhibitory factors on inflammatory bone loss. However, it is worth mentioning that the introduction of excessive inhibitory cytokines and cell subsets may also destroy local tissue homeostasis, leading to inflammation, which is similar to the effect of “keystone” pathogens. In summary, the periodontal-specific cytokine network and host immune response are worthy of further investigation. Research progress in this field may contribute to the development of tissue-specific therapeutic technology and reductions in the burden to periodontal patients and society and the influence of local periodontal inflammation on related systemic diseases.
